# The influence of grandmothers on breastfeeding rates: a systematic review

**DOI:** 10.1186/s12884-016-0880-5

**Published:** 2016-04-27

**Authors:** Joel Negin, Jenna Coffman, Pavle Vizintin, Camille Raynes-Greenow

**Affiliations:** Sydney School of Public Health, University of Sydney, Edward Ford Building (A27), Sydney, NSW 2006 Australia

**Keywords:** Grandmother, Mother-in-law, Breastfeeding, Exclusive breastfeeding

## Abstract

**Background:**

Exclusive breastfeeding for the first six months of an infant’s life has enormous potential to reduce mortality and morbidity. The older generation, particularly the infant’s grandmothers, play a central role in various aspects of pregnancy and child rearing decision-making within the family unit. This is particularly true in low- and middle-income countries where older women are seen as owners of traditional knowledge. Despite this, most health programs target the individual person most directly involved in the target behaviour – usually new mothers – without a commensurate understanding of who else influences those decisions. In this systematic review we aim to quantify the impact of the grandmother on influencing a mother’s breastfeeding practices.

**Methods:**

We conducted a systematic review using Web of Science, Scopus, and Medline databases using search terms for grandmother and breastfeeding. Eligible studies reported on the duration of exclusive breastfeeding and included estimates of effect of a grandmother’s influence including whether or not the grandmother lived with the infant’s family, the grandmother’s education, and the grandmother’s attitudes towards and prior experience with breastfeeding.

**Results:**

We identified 568 articles and, after review, 13 articles were assessed as meeting the selection criteria. They were conducted in both developed and developing countries and included cross-sectional surveys, prospective cohort studies and one randomised controlled trial. Eight studies examined the effects of attitudes or experiences of older generations with respect to breastfeeding and five of the eight found a significant positive impact on breastfeeding when grandmothers of the infants had had their own breastfeeding experience or were positively inclined towards breastfeeding, resulting in effects of between 1.6 to 12.4 times more likely to exclusively breastfeed or refrain from introducing solid foods. A Chinese study however found that highly educated grandmothers were associated with decreased exclusive breastfeeding. The majority of the studies were assessed to be of weak or moderate quality.

**Conclusions:**

This review found evidence that demonstrates that grandmothers have the capacity to influence exclusive breastfeeding. Programs that seek to influence exclusive breastfeeding should include grandmothers in their interventions to achieve maximum impact.

## Background

Exclusive breastfeeding for the first six months of an infant’s life has the greatest potential to reduce mortality of all preventive interventions with an estimated potential to avert over 800,000 deaths, or 13 % of all deaths in children under five in the developing world [1–3]. Not only does exclusive breastfeeding have the potential to directly avert deaths in children under-five, but evidence also suggests that it has an indirect protective effect against gastrointestinal infections, respiratory infections, allergic diseases and non-transmissible chronic diseases that appear later in life, such as obesity, diabetes, Crohn’s disease, and lymphoma [4]. Despite the clear benefits and numerous public health campaigns to promote breastfeeding, exclusive breastfeeding rates are low. According to UNICEF, the global average of exclusive breastfeeding in infants under six months of age is 41 %, with the lowest rate in low- and middle-income countries being 25 % in the West and Central Africa region [5]. High income countries have even lower rates of exclusive breastfeeding: in the United States, only 16 % of infants are exclusively breastfed at six months [6] and, in Australia, only 15 % at five months [7].

Breastfeeding rates are influenced by a myriad of factors spanning from sociocultural to economic. Literature suggests that the older generation, particularly the infant’s grandmothers (either the maternal mother or the paternal mother), play a central role in various aspects of pregnancy decision-making and child rearing within the family unit [8–10]. This is particularly true in low- and middle-income countries. For example, in Tanzania, a study found that paternal grandmothers considered themselves responsible for all family health care issues and actively discouraged prevention of mother-to-child HIV transmission practices [11]. Similarly, a Nepalese study among mothers-in-law noted that they “see themselves as key providers of, and decision-makers in, perinatal care practices” [12]. A female respondent in a Ghanaian study on stated that she would only go to the clinic if her baby was sick with “my mother-in-law’s permission” [13]. Within certain contexts, evidence indicates that this influence can be particularly deleterious to exclusive breastfeeding rates. A 2012 study from Nigeria, found that paternal grandmothers pressured 25 % of the mothers enrolled in the study to not exclusively breastfeed [14].

In many societies around the world, older women are seen as owners of traditional knowledge and cultural history which has strong community significance [15]. Despite this influence, the older generation has not been a specific area of study in the field of global health [16]. Most health programs target the individual person most directly involved in the target behaviour – usually new mothers [17, 18] – without a commensurate understanding of who else influences those decisions.

In this systematic review we aim to quantify the impact of the grandmother on influencing a mother’s breastfeeding practices.

## Methods

We adhered to the PRISMA guidelines for systematic reviews [19]. The search terms “mother-in-law”, “mothers-in-law”, “grandmother” and “grandmothers” were combined with the terms “breastfeeding” and “breast-feeding” and entered into the Web of Science, Scopus, and Medline databases.

We defined grandmothers as the mother of either the mother or the father of the infant whose feeding outcome is being measured. We defined (exclusive) breastfeeding according to the WHO’s definition of exclusive breast feeding as “no other food or drink, not even water.” Eligible studies reported on the duration of exclusive breastfeeding. Eligible studies also included estimates of effect of a grandmother of the infant’s duration of exclusive breastfeeding, and included a sample size greater than 50 – studies under this size did not have the power to detect a difference in rates. Qualitative studies were excluded. We limited the search to publications in English from 1995 until 2014 to leverage 20 years of evidence since the target implementation date of UNICEF’s [20].

In the first review round, two reviewers (PV, JC) independently scrutinised the list of article titles and eliminated the clearly non-relevant titles. Another round of exclusions using abstracts of those titles deemed potentially relevant was conducted and the final selection was based on the full text of potentially relevant abstracts. In cases of disagreement, a third reviewer (JN) examined the articles. Results and inclusion were discussed until consensus was reached among all three reviewers.

Data were then extracted from each relevant study into an excel spreadsheet. Extracted information included year, location of study, brief description, study type, sample size, study population, review of methods, primary outcome measure and focus topic of the study. Quality was assessed according to the Effective Public Health Practice Project Quality Assessment Tool for Quantitative Studies [21] which gives a rating from between ‘poor’ to ‘strong’ based on a range of criteria including selection bias, study design, confounders, blinding, data collection methods, withdrawals and drop-outs, intervention integrity and analyses. The tool has been assessed for validity and reliability and was recommended for use by Deeks and colleagues [22].

The reported measure of effect (e.g. odds ratio, hazard ratio) from each study was used to compare across studies with the same type of measurement. Diverse elements of grandmother influence were assessed including co-habitation status (whether or not the grandmother lived with the infant’s family), the grandmother’s education, and the grandmother’s attitudes towards and prior experience with breastfeeding. For each element of influence, a narrative synthesis is provided. Figures are provided to show the effect of the grandmother. Figures are presented separately by measure of effect and data is not combined across studies.

Ethical approval was not required for this study.

## Results

**Fig. 1:**
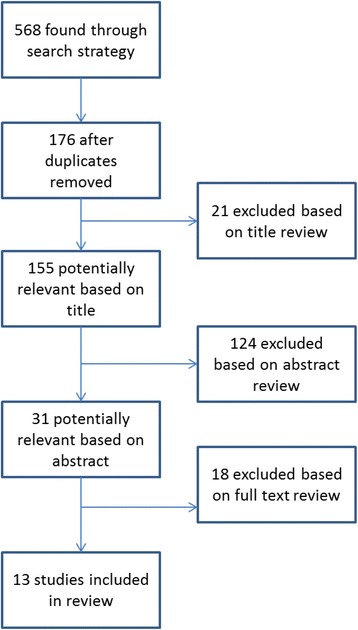
Selection of manuscripts for systematic review of impact of older generation on breastfeeding

The searches yielded 568 journal articles in total (Fig. 1). Following the removal of duplicates, 176 articles remained, of which 155 were potentially relevant based on a review of the title. A further 124 articles were excluded from an abstract review; 7 articles had a sample population of less than 50; 4 articles were not in English; 50 were unrelated to either breastfeeding or the influence of grandmothers; and 62 were either small qualitative studies or narratives. Of the 31 potentially relevant abstracts, 13 articles were included after full-text review due to inclusion of estimates of effect which could be compared across studies (Fig. 1).

Once the 13 articles to be included in the review were determined we performed a quality analysis using the Effective Public Health Practice Project *Quality Assessment Tool for Quantitative Studies* [21]. Two reviewers independently assessed each study according to the Quality Assessment Tool and then discussed and reviewed until consensus of the final quality assessment.

Thirteen studies were included (Table 1). The studies were conducted in a range of countries, which included both low and high-income countries. Of these, there were eight cross-sectional surveys [23–29], four prospective cohort studies [4, 30–32] and one randomised controlled trial [33]. Studies included a range of time points of exclusive breastfeeding from one week to six months. Of the thirteen studies, eight assessed the impact the grandmother has on breastfeeding rates and duration due to her attitude to or experience of breastfeeding [23–26, 28–31]. Two studies assessed the impact on breastfeeding rates and duration when the grandmother was the main childcare taker [27, 32]. One study assessed the impact the grandmother’s education level had on breastfeeding rates and duration.

**Table 1:** Characteristics of included studies

Author	Year	Title	Country	Study design/method	Sample size	Outcome measurement used	Effect measurement	Impact of the grandmother (measurement)	Study quality (according to the EPHPP assessment tool) [21]
Chen, T. L., et al. [23]	2011	Cultural factors and social support related to breastfeeding among immigrant mothers in Taipei City, Taiwan	Taiwan	Cross-sectional survey/questionnaire	210	Exclusive and partial breastfeeding at 3 months postpartum	Relative risk	Grandmother’s own experience with breastfeeding	Moderate
Dashti, M., et al. [24]	2014	Predictors of breastfeeding duration among women in Kuwait: results of a prospective cohort study	Kuwait	Cross-sectional survey/Questionnaires	373	Exclusive and partial breastfeeding at 6 months postpartum	Hazard ratio	Grandmother’s EBF preference	Moderate
Duong, D. V., et al. [26]	2005	Introduction of complementary food to infants within the first six months postpartum in rural Vietnam	Vietnam	Cross-sectional survey/Household surveys	2690	Exclusive breastfeeding at 1 week, 4 months and 6 months postpartum	Odds ratio	Grandmother’s EBF preference	Weak
Duong, D. V., et al. [25]	2004	Breast-feeding initiation and exclusive breast-feeding in rural Vietnam	Vietnam	Cross-sectional survey Questionnaires	463	Exclusive breastfeeding at 1 week postpartum	Odds ratio	Grandmother’s EBF preference	Weak
Kohlhuber, M., et al. [30]	2008	Breastfeeding rates and duration in Germany: a Bavarian cohort study	Germany	Prospective cohort study Questionnaires	3822	Exclusive and partial breastfeeding at 2, 4 and 6 months postpartum	Odds ratio	Attitude towards breastfeeding by grandmother	Moderately weak
Li, Y., et al. [27]	1999	Breast-feeding in Bangkok, Thailand: Current status, maternal knowledge, attitude and social support	Thailand	Cross-sectional survey Questionnaires	221	Exclusive and partial breastfeeding at 3 months postpartum	Odds ratio	Where grandmother is main caregiver	Weak
Liu, J. H., et al. [34]	2013	Social and demographic determinants for breastfeeding in a rural, suburban and city area of South East China	China	Cross-sectional survey Questionnaires	1385	Exclusive and partial breastfeeding at 6 months postpartum	Odds ratio	Level of education of grandmother	Moderate
Ludvigsson, J. F.[28]	2003	Breastfeeding in Bolivia - Information and attitudes	Bolivia	Cross-sectional survey/interviews	502	Exclusive and partial breastfeeding up to 12 months postpartum	Odds ratio	Attitude towards EBF by grandmother	Moderately weak
Mahoney, M. C. and D. M. James [29]	2000	Predictors of anticipated breastfeeding in an urban, low-income setting	U.S.A	Cross-sectional survey/survey	66	Anticipated exclusive and partial breastfeeding ranging from 4 weeks to 52 weeks postpartum	Relative risk	EBF encouragement by baby’s father or the maternal grandmother	Weak
Nunes, L. M., et al. [33]	2011	Reduction of Unnecessary Intake of Water and Herbal Teas on Breast-fed Infants: A Randomized Clinical Trial With Adolescent Mothers and Grandmothers	Brazil	Randomised control trial	323	Exclusive breastfeeding at 6 months postpartum	Hazard ratio	Cohabitation with maternal grandmother	Strong
Santo, L. C. D. E., et al. [4]	2007	Factors associated with low incidence of exclusive breastfeeding for the first 6 months	Brazil	Prospective cohort study/Questionnaires	220	Exclusive breastfeeding at 6 months postpartum	Hazard ratio	Cohabitation with maternal or paternal grandmother	Moderate
Susin, L. R. O., et al. [31]	2005	Influence of grandmothers on breastfeeding practices	Brazil	Prospective cohort study/Questionnaires	601	Exclusive breastfeeding at 6 months postpartum	Odds ratio	(a) Frequency of contact with maternal or paternal grandmother; (b) where maternal or paternal grandmother advises other liquids	Moderate
Wasser, H. M., et al. [32]	2013	Who’s feeding baby? Non-maternal involvement in feeding and its association with dietary intakes among infants and toddlers	U.S.A	Prospective Cohort study/Questionnaires	209	Non-maternal feeding of the infant at 3,6, 9, 12 and 18 months	Odds ratio	Where grandmother is main caregiver	Weak

The included studies used a variety of measures of effect including odds ratios, relative risks and hazard ratios. Two studies used relative risk as the measure of effect, eight studies used odds ratios, and three studies used hazard ratios, therefore we were unable to calculate an overall effect size. The majority of the studies were of weak or moderate quality, according to the Effective Public Health Practice Project *Quality Assessment Tool for Quantitative Studies* [21].

**Fig. 2:**
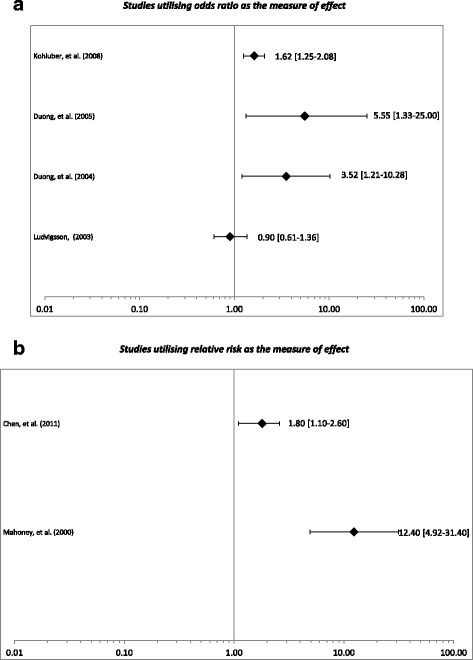
**a** Impact on breastfeeding due to the grandmothers’ positive attitude towards breastfeeding. *Studies utilising odds ratio as the measure of effect*. **b** Impact on breastfeeding due to the grandmothers’ positive attitude towards breastfeeding. *Studies utilising relative risk as the measure of effect*

The types of influences grandmothers had on breastfeeding differed from study to study, as some measured the older generation’s personal experience with breastfeeding, whilst others measured their attitudes towards breastfeeding. Of the 13 studies examined in this review, eight examined the effects of attitudes or experiences of grandmothers with respect to breastfeeding [23–29, 31]. A majority of these (five of eight) found that there was a significant positive impact on breastfeeding when grandmothers of the infants had had their own breastfeeding experience or were positively inclined towards breastfeeding, resulting in effects of between 1.6 to 12.4 times more likely to exclusively breastfeed or refrain from introducing solid foods (Fig. 2) [23–26, 29]. Two studies found no significant effect on breastfeeding rates from grandmothers who had positive attitudes to breastfeeding [28, 32].

**Fig. 3:**
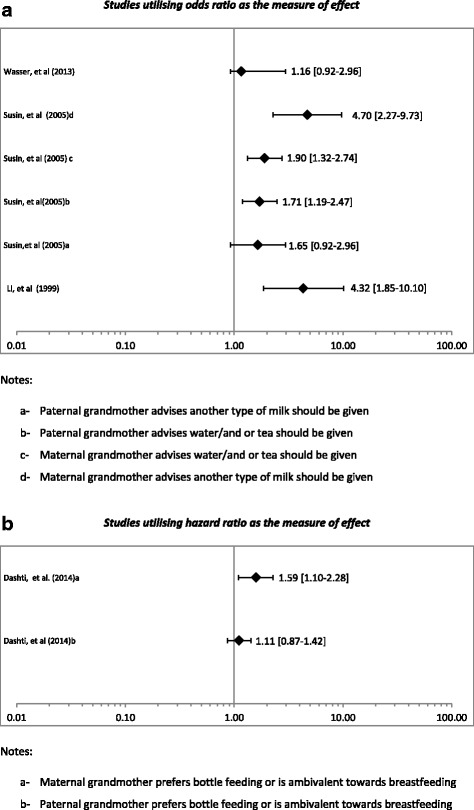
**a** Impact on discontinuation of breastfeeding due to the grandmother’s negative attitude towards breastfeeding. *Studies utilising odds ratio as the measure of effect*. **b** Impact on discontinuation of breastfeeding due to the grandmother’s negative attitude towards breastfeeding. *Studies utilising hazard ratio as the measure of effect*

Three of the thirteen studies reported negative impacts of grandmothers on breastfeeding. Kohlhuber et al. [30] found within a sample population in Germany, if a maternal grandmother had a negative attitude towards breastfeeding, the mother was up to 3.62 (95 % CI 2.26,5.81) times more likely not to initiate breastfeeding after birth. Similarly, Susin et al. [31] found that when the maternal grandmother advised giving the infant water or tea, the mother was 2.22 (95 % CI 1.5, 3.30) times more likely to abandon exclusive breastfeeding by the end of the first month. Li et al [27] found that if the grandmother was the primary caretaker of the infant, the mother was up to 4.3 (95 % CI 1.85-10.10) times more likely to practice non-exclusive breastfeeding (Fig. 3).

Of the 13 studies included in this review, there was only one study that involved a randomised controlled trial. Nunes et al. [33] investigated the effect of an intervention aimed at grandmothers and adolescents to reduce tea and water intake of infants less than six months of age in Brazil. The study measured the introduction of water and/or herbal teas over the first six months of the infants’ life among two groups; cohabiting grandmothers and mothers, and mothers and grandmothers that did not live together. There was a reduced chance of exclusive breastfeeding among women who cohabited with grandmothers than those who lived apart from their grandmothers.

**Fig. 4:**
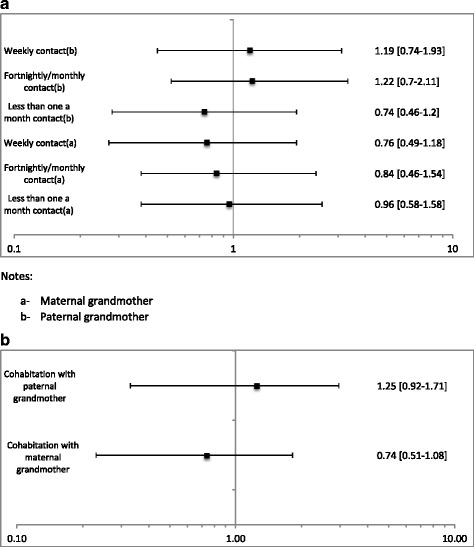
**a** Effect of cohabitation with grandmother on exclusive breastfeeding. **b** Effect of cohabitation with grandmother on exclusive breastfeeding

Findings from other studies investigating the impact of cohabitation with grandmothers on exclusive breastfeeding are less clear. Susin et al. [31] investigated the frequency of contact of grandmothers with mothers, but did not find that grandmothers had a significant influence on breastfeeding, irrespective of the length of contact with mothers (Fig. 4a). Similarly, Santo et al. [4] investigated the impact of the maternal compared to the paternal grandmother cohabitating with the mother on exclusive breastfeeding. The study did not find a significant influence of cohabitation with either grandmother upon breastfeeding (Fig. 4b).

Only one study specifically examined the impact of the grandmother’s educational level on exclusive breastfeeding. Liu et al. [34] compared grandmothers with a formal education compared to no formal education and found that mothers were significantly less likely to exclusively breastfeed if the grandmothers were educated. Liu suggests that the fact that highly educated grandmothers were associated with decreased exclusive breastfeeding may reflect the relationship between better family socioeconomic status and preference to formula feed due to formula being seen as indicative of higher socioeconomic status.

## Discussion

This review found evidence that demonstrates that grandmothers have the capacity to influence exclusive breastfeeding. Although there were differences in the type of breastfeeding outcome and how the grandmothers influence was measured, the overall effect on breastfeeding was positive when the older female generations’ attitudes towards or experiences with breastfeeding was favourable towards breastfeeding. A grandmother’s positive breastfeeding opinion had the potential to influence a mother up to 12 % more likely to initiate breastfeeding. Conversely a negative opinion has the capacity to decrease the likelihood of breastfeeding by up to 70 %.

Whilst this review has shown that the grandmother can have a significant impact on exclusive breastfeeding, there is still insufficient research on this topic to empirically state to what extent their impact has and what other factors contribute to their influence. The impact of cohabitation with grandmothers on the breastfeeding rates of mothers was unclear in the two studies that investigated this factor. The associations between duration of contact between the grandmother and mother and breastfeeding rates were not statistically significant, as was the association of cohabitation with either maternal or paternal grandmothers. As both studies took place in a similar region within Brazil, to infer that cohabitation has no effect on breastfeeding rates would ignore the potential for an effect to exist within other contexts.

One of the key limitations of this study is the difficulty in accurately comparing the studies due to the heterogeneity of the effect measurements used. The authors did attempt to mitigate this problem by contacting a number of authors in order to obtain the original data from each study; however, we did not receive any correspondence.

Another major limitation of this study, therefore, is the cross-cultural generalisability of these findings. Experiences of mothers being influenced by the infant’s grandmother in a high-income country such as the United States might be dramatically different from a country with a lower income such as Pakistan [35–37]. The finding by Liu et al. that mothers that had a grandmother with an education were less likely to exclusively breastfeed may have more to do with the country-specific, highly targeted infant formula advertising in China, rather than an effect of education itself [38]. Furthermore, cultural and socioeconomic differences in ethnic groups within the same country would have the potential to have different influences on breastfeeding practices. For example, Yoruba and Edo communities in Nigeria tend to consider exclusive breastfeeding hazardous to an infant’s health, whilst other communities might not share the same beliefs [39].

## Conclusion

This review suggests that grandmothers can have an influence in one of the most important health behaviours. Results of this review suggest that female members of the older generation exert influence on breastfeeding, and researchers and program developers can use these findings to include this influence in their own intervention or program developments. Some commentators have started to acknowledge this need. One notes that “programs must be sensitive to social expectations… and involve mothers-in-law… in counselling of mothers who intend to practice [exclusive breastfeeding]” [40] and another states that “managers of communications campaigns should consider directly targeting mothers-in-law to increase their support for the use of family planning” [41].

The lack of attention to mothers-in-law in global health fits in to a larger reality of the neglect of older adults by the global health community. Global health funding and practice has largely focused on mothers and children at the expense of not just men [42] but, even more so, older adults [43, 44]. This represents a gap in global health practice and health promotion in particular. Of greater concern is the suggestion that the perspectives of older women are met with derision by some health workers which has the potential of only consolidating their traditional practices and rejection of new practices [15].

There are, however, recent research projects that take a more inclusive approach. An ongoing Indian randomised controlled trial has involved mothers-in-law in efforts to reduce domestic violence [45]. Programmes involving the older generation focused on breastfeeding could have an important impact. Engaging older women with health messages can improve their own health outcomes, those of their daughters and daughters-in-law as well as their grandchildren. Very few public health interventions can claim such cross-generational impact.

### Availability of data and materials

All data found in this systematic review is available through the database searches outlined in the methods section of the manuscript.
